# Association of functional dorsal attention network alterations with breast cancer and chemotherapy

**DOI:** 10.1038/s41598-018-36380-6

**Published:** 2019-01-14

**Authors:** Chao-Yu Shen, Vincent Chin-Hung Chen, Dah-Cherng Yeh, Shu-Ling Huang, Xuan-Ru Zhang, Jyh-Wen Chai, Yen-Hsun Huang, Ming-Chih Chou, Jun-Cheng Weng

**Affiliations:** 1 Institute of Medicine, Chung Shan Medical University, Taichung, Taiwan; 2 School of Medicine, Chung Shan Medical University, Taichung, Taiwan; 3 Department of Medical Imaging, Chung Shan Medical University Hospital, Taichung, Taiwan; 4 School of Medicine, Chang Gung University, Taoyuan, Taiwan; 5 Department of Psychiatry, Chang Gung Memorial Hospital, Chiayi, Taiwan; 6 Breast Medical Center, Cheng Ching Hospital Chung Kang Branch, Taichung, Taiwan; 7 Department of Psychology, Chung Shan Medical University, Taichung, Taiwan; 8 Department of Radiology, Taichung Veterans General Hospital, Taichung, Taiwan; 9 College of Medicine, China Medical University, Taichung, Taiwan; 10 Department of Child and Adolescent Psychiatry, Taipei City Psychiatric Center, Taipei City Hospital, Taipei, Taiwan; 11 Division of Thoracic Surgery, Department of Surgery, Chung Shan Medical University Hospital, Taichung, Taiwan; 12 Department of Medical Imaging and Radiological Sciences, Chang Gung University, Taoyuan, Taiwan

## Abstract

Breast cancer is the most common cancer among women worldwide. Adjuvant chemotherapy has significantly reduced mortality but increased cognitive impairments, including attention function, making quality of life issues a crucial concern. This study enrolled nineteen breast cancer patients who were treated with standard chemotherapy within 6 months and 20 sex-matched healthy controls to investigate the brain effects of chemotherapy. All participants underwent resting-state functional magnetic resonance imaging (rs-fMRI) with mean fractional amplitude of low-frequency fluctuation (mfALFF) analysis and were correlated with neuropsychological tests, including the Mini-Mental State Examination (MMSE), the Cognitive and Affective Mindfulness Scale-Revised (CAMS-R), and the Impact of Event Scale-Revised (IES-R), to explore the possible underlying mechanism of cognitive alternations. We found increased mfALFF over the frontoparietal lobe and decreased mfALFF over the occipital lobe in the cancer patients compared with the healthy controls; the altered brain regions may be associated with the dorsal attention network (DAN) and may be explained by a compensatory mechanism. Both MMSE and CAMS-R scores showed a positive correlation with mfALFF in the occipital lobe but a negative correlation in the frontoparietal lobe. By contrast, IES-R scores showed a positive correlation with mfALFF in the frontoparietal lobe but a negative correlation in the occipital lobe. These alterations are potentially related to the effects of both chemotherapy and psychological distress. Future research involving a larger sample size of patients with breast cancer is recommended.

## Introduction

Breast cancer is the most common cancer among women worldwide, and according to the American Cancer Society, it accounts for 25% of all new cancer diagnoses in women globally. In addition, there were approximately 250,000 new breast cancer diagnoses among women and more than 3.5 million women living with a history of breast cancer in the United States in 2017^1,2^. Adjuvant chemotherapy significantly minimized the recurrence risk of breast cancer, and different chemotherapy regimens are estimated to be responsible for a reduction of 35%-60% in the mortality^3^. However, many studies have reported cognitive impairments following the process of adjuvant chemotherapy in breast cancer survivors^4–7^. The increased incidence of breast cancer, coupled with a longer survival time, has resulted in a high number of breast cancer survivors who are struggling with cognitive impairments. Quality of life issues are becoming crucial considerations for patients with breast cancer, physicians, and researchers.

The term ‘chemobrain’ was created to describe cognitive impairments experienced by patients after receiving chemotherapy^7–13^. Typical concerns reported by patients with ‘chemobrain’ include difficulty concentrating or staying focused on a task, trouble remembering details, difficulty multitasking, slower processing speeds, and memory lapses^13^. Wefel *et al*. reviewed 53 published cross-sectional and prospective neuropsychological studies that provided mixed evidence of chemotherapy-related cognitive dysfunction in women treated for breast cancer and found that attention, memory, processing speed, and executive function were the most commonly affected cognitive domains^12^. These effects appear to be more pronounced in the short term; however, Koppelmans *et al*. recently revealed that breast cancer survivors exhibited a poorer performance in neuropsychological tests 20 years after they had undergone chemotherapy than their matched healthy controls. Thus, the effects of chemotherapy on the brain may be long lasting^14^. However, recent researches have revealed that cognitive alterations, including compromised attention and working memory, can exist in patients with breast cancer before undergoing adjuvant chemotherapy, indicating psychological distress (due to the general effects of cancer diagnosis and local treatment) as a possible contributor to cognitive dysfunction^15–18^. Compared with healthy controls, Hermelink *et al*. revealed that patients with breast cancer, both before and 1 year after chemotherapy, showed cognitive decline and lower accuracy in attention tests. The effect of attention alteration was significantly associated with the effect of post-traumatic stress disorder (PTSD)^19^. Taken together, these studies suggest that psychological factors play an important role in breast cancer-related cognitive impairments rather than the effects of chemotherapy alone.

Evaluating the effect of cancer treatment on cognitive abilities usually involves objective neuropsychological testing and subjective (questionnaire) evaluation. Objective testing assesses multiple cognitive domains and has the sensitivity to detect subtle changes following the process of cancer treatment. Subjective (questionnaire) testing assesses problems associated with depression, anxiety, and fatigue. However, the subjective evaluation of cognitive dysfunction may not satisfactorily correlate with objective neuropsychological testing, resulting in many unanswered questions regarding cancer-related cognitive deficits and the underlying neural bases^20–22^.

Recently, neuroimaging methods have been used to examine the effects of breast cancer and its associated treatments on brain structures to understand the underlying processes contributing to cognitive changes. In a recent study using magnetic resonance imaging (MRI) voxel-based morphometry, a method used to quantitatively evaluate brain structure changes on a voxel-by-voxel basis in three-dimensional (3D) MRI, the authors found that the regional gray matter density, including the bilateral frontal, temporal, and cerebellar regions and the right thalamus, declined in patients with breast cancer from baseline to 1 month after chemotherapy. However, recovery was observed in some regions at l year after chemotherapy^23^. By using diffusion tensor imaging (DTI) analysis, a noninvasive method that provides unique microstructural information of the brain white matter tissue, Deprez *et al*. found significantly decreased brain fractional anisotropy (FA) in the frontal, parietal, and occipital white matter tracts, which correlated with attention and verbal memory performance changes in patients with breast cancer 3 to 4 months after chemotherapy treatment when compared with the baseline^24^. In recent review studies, Kaiser *et al*. and Pomykala *et al*. concluded that structural brain imaging, including volumetric and DTI analyses, revealed both reduced gray and white matter volume and decreased white matter integrity from months to years after chemotherapy, but no difference was found between breast cancer patients before chemotherapy and healthy controls. It supports the notion that structural brain differences that are found after chemotherapy are indeed the result of exposure to chemotherapeutic agents^25,26^.

In addition to structural approaches, MRI can be used to measure brain activity by detecting blood oxygen level-dependent (BOLD) signal changes in blood flow of the brain through functional MRI (fMRI). fMRI can assess brain activities and functional connections that are believed to be more sensitive than structural MRI in detecting brain cognitive function and has been used to evaluate several clinical populations with cognitive impairments, including those with Alzheimer’s dementia, schizophrenia, traumatic brain injury, and attention deficit hyperactivity disorder (ADHD)^27–30^. fMRI can generally be deviated into task-based and resting-state (task-free) methods. Task-based fMRI studies are designed with stimulus protocols during the MRI scan, and brain activities in response to these stimuli are analyzed. The majority of task-based fMRI studies in breast cancer (e.g., visual memory, verbal memory, attention, and executive functioning tasks) have revealed that breast cancer survivors displayed alterations of multifocal cerebral cortical activities and functional networks, which differed according to task designs^31–35^. By using task-based fMRI, McDonald *et al*. found decreased working memory-related activation in the frontal lobe in patients with breast cancer 1 month after chemotherapy that partially recovered at 1 year^32^. Additionally, Dumas *et al*. found decreased functional connectivity in patients with breast cancer 1 month after chemotherapy that partially returned to baseline at 1 year in the dorsal attention network, but decreased connectivity was observed in the default mode network at 1 month and 1 year following chemotherapy^35^. However, successful execution of the task-based fMRI is highly dependent on the task design or difficulty and a participant’s cooperation. By contrast, a resting-state (task-free) method, a so-called resting-state fMRI (rs-fMRI), simplifies the examination of brain activity during the resting status of participants through highly standardized and reproducible procedures^36–38^. Therefore, rs-fMRI has attracted attention from researchers interested in widening its use in clinical applications. By using rs-fMRI with seed-based correlation analysis (SCA), Miao *et al*. revealed decreased functional connectivity in the dorsal medial prefrontal cortex and medial temporal lobe subsystems and may be associated with the attention function of breast cancer patients 1 month after chemotherapy^39^. Similarly, Wang *et al*. revealed decreased functional connectivity in the dorsolateral prefrontal cortex and inferior frontal gyrus, which correlated with executive deficits of breast cancer patients 1 month after chemotherapy^40^. Miao *et al*. also found decreased functional connectivity in the anterior cingulate cortex in breast cancer patients 3 years after chemotherapy, which correlated with executive function impairment^41^. However, the main advantage of SCA is being able to ask a straightforward question about connectivity and receive a direct answer but within the limit of being able to frame the original question by means of a well-defined seed^42^. Using rs-fMRI with graph theoretical analysis (GTA), based on topology and allowing for the segregation and integration of the connectome information^43,44^, Xuan *et al*. revealed abnormal organization of large-scale functional brain networks of both global and local efficiency in patients with breast cancer 1 month after undergoing chemotherapy^45^. Similarly, Bruno *et al*. found significantly decreased global clustering as well as disrupted regional network characteristics in the frontal, striatal, and temporal areas in breast cancer survivors 5 years after undergoing chemotherapy. Moreover, breast cancer survivors showed significantly increased self-reporting of executive function and memory difficulties compared with healthy controls^46^. Although connectome analyses can improve the characterization of the brain’s complexity, it can be computationally intensive, and thresholding networks of different sizes for comparison remain an area of ongoing debate^47^. The mean fractional amplitude of low-frequency fluctuations (mfALFF), which is based on frequency-domain analysis, is considered to be physiologically meaningful and reflective of spontaneous neural activity^48,49^. It may thereby provide a useful complement to approaches, such as interregional coherencies between multiple BOLD signals^42^.

The majority of rs-fMRI studies regarding breast cancer have focused on the short- and long-term effects of chemotherapy, and whether the evidence from objective neuropsychological testing, subjective (questionnaire) evaluation, and neuroimaging studies can be satisfactorily correlated with the assessment of chemotherapy effects and the possible psychological distress of breast cancer remains unclear. Therefore, to address the lack of research, this study investigated patients with breast cancer within 6 months after chemotherapy by using rs-fMRI with mfALFF analysis and correlated them with objective and subjective neuropsychological testing results to explore the possible underlying mechanism of cognitive alternations in breast cancer survivors.

## Materials and Methods

### Participants

Participants were recruited from the Taichung Veterans General Hospital in Taichung, Taiwan. Inclusion criteria for patients with breast cancer were female sex, age between 20 and 55 years old, and histological confirmation of primary breast cancer within 6 months after completion of chemotherapy with standard chemotherapeutic agents (docetaxel and epirubicin). The exclusion criteria for patients with breast cancer were being in a terminal stage of the disease (defined as a life expectancy of less than 1 year), having a history of treatment for any cancer other than breast cancer or double cancer, undergoing radiation therapy before the investigation, having an indication of brain metastasis on postcontrast MRI findings or any known brain lesion from previous brain image studies, having any contraindication of MRI scans, and having a history of psychiatric or neurological illnesses or substance-use disorders. Aged-matched healthy women were recruited as healthy controls. The exclusion criteria for healthy controls were a history of psychiatric or neurological illnesses or substance-use disorders, a family history of major psychiatric or neurological illnesses, a current prescription of any psychiatric or psychotropic medications, current pregnancy or breastfeeding, and any contraindication of MRI scans. The study was approved by the Institutional Review Board of the Chung Shan Medical University Hospital (No. CS13121) and the Taichung Veterans General Hospital (No. SF14185A). All participants participated in the study after providing informed consent, and all research was performed in accordance with relevant guidelines and regulations.

### Objective Neuropsychological Testing and Subjective (Questionnaire) Evaluation

All participants were evaluated by a trained psychotherapist using the structured diagnostic interview of the fifth edition of the Mini-International Neuropsychiatric Interview^50^. The Mini-Mental State Examination (MMSE) was used for objective neuropsychological testing in this study because it has been extensively used in research and clinical practice to assess cognitive function^51^. The MMSE was also adopted as a screening tool to assess cognitive dysfunction in cancer survivors and monitor changes in cognitive status resulting from treatments^52^. The MMSE features the items of orientation to time and place, registration of words, attention, calculation, and word recall, as well as language and visual construction. Scores range from 0 to 30, with a higher score indicating a higher cognitive function. The subjective (questionnaire) evaluations used in this study were the Cognitive and Affective Mindfulness Scale-Revised (CAMS-R) and the Impact of Event Scale-Revised (IES-R). The CAMS-R is a 12-item measure designed to capture a broad conceptualization of mindfulness with language that is not specific to any particular type of meditation training. Higher values reflect greater mindful qualities^53^. The IES-R is a 22-item self-report measure that assesses subjective distress caused by traumatic events. Respondents are asked to identify a specific stressful life event and then indicate how much they were distressed or bothered during the past 7 days by each ‘difficulty’ listed^54^.

### MRI Scan Acquisition

All participants’ MRI examinations were performed on a 1.5 T MRI scanner (Magnetom Aera, Siemens Medical Systems, Erlangen, Germany) with a standard 8-channel head coil. T1-weighted 3D volume images were acquired with a magnetization prepared rapid gradient echo sequence (TR/TE/TI/FA = 9.11 ms/1.77 ms/450 ms/7°; 124 axial slices; and voxel size = 1.0 × 1.0 × 1.4 mm^3^). For functional MRI measurements during the resting-state condition, a total of 256 volumes were recorded with a temporal resolution of 2 s using a gradient echo-echo planar imaging sequence with 33 axial slices per volume (TR/TE/FA = 2000 ms/30 ms/90°; voxel size = 3.4 × 3.4 × 4.0 mm^3^). The participants were instructed to lie down, remain motionless, keep their eyes closed, relax, think of nothing in particular, and remain awake during this examination. Cushions and earmuffs were used to reduce motion and scanner noise for participants. Other MRI pulse sequences, including axial T1WI, T2WI, fluid-attenuated inversion recovery (FLAIR), and coronal T2WI, were performed in both breast cancer patient and healthy control groups, and additional postcontrast axial, coronal and sagittal T1WI were performed in breast cancer patients to rule out the possibility of a brain metastasis.

### Resting-State Functional MRI Data Preprocessing and Analysis

Preprocessing was conducted using Statistical Parametric Mapping 8 (SPM8, Wellcome Department of Cognitive Neurology, London, UK) based on MATLAB 2013 (Math-Work, Natick, MA, USA). After slice-timing correction, we calculated the center of each image and realigned the data to the first volume for motion correction. Following motion correction, data were normalized to the Montreal Neurological Institute (MNI) standard space with an affine transformation and resampled to isotropic 3-mm voxels. The data were then spatially smoothed using a 6-mm full width at half maximum Gaussian kernel for a better signal-to-noise ratio gain. Nuisance regression was then performed using six head motion parameters as covariates. Then, the whole brain, white matter, and cerebrospinal fluid masks were used to remove the physiological noise. Linear detrending and bandpass temporal filtering were performed on the time series of each voxel to minimize the effects of low-frequency drifts and physiological signals by using the Resting-State Data Analysis toolkit v1.8 (REST v1.8, Center for Cognition and Brain Disorders, Hangzhou Normal University, Zhejiang, China). According to studies’ suggestions and our study experience, we set the frequency range from 0.01 to 0.12 Hz to include important physiological information and mitigate the influence of low-frequency drift and high-frequency physiological noise^37,48,55,56^. The mean fractional amplitude of low-frequency fluctuations (mfALFF) analysis was calculated in the frequency range of 0.01 to 0.12 Hz, and for a given voxel, the time series was first converted to the frequency domain using a fast Fourier Transform. The square root of the power spectrum was computed, averaged, and normalized across a predefined frequency interval, which was termed the ALFF at the given voxel^49^. We then performed two-sample t-tests to assess the difference in mfALFF between the patients with breast cancer and healthy controls. To investigate the relationship between the mfALFF and cognitive tests, we calculated the correlation between mfALFF and MMSE, CAMS-R, and IES-R of all the participants with multiple regressions by SPM8. In addition, age and years of education were used as covariates, and a false discovery rate (FDR) corrected p-value of less than 0.05 was considered significant. To view the results, we used a T1-weighted MNI template to create the underlying map. The datasets analyzed during the current study are available from the corresponding author on reasonable request.

## Results

### Study Participants and Cognitive Tests

A total of 39 participants were recruited and enrolled: 19 female patients with breast cancer age range: 32 to 55 years, mean age: 43.8 ± 6.4 years; years of education: 13.9 ± 2.2; breast cancer stage: I (n = 2), II (n = 14), III (n = 3) and 20 healthy female controls (age range: 43 to 55 years, mean age: 50.1 ± 2.5 years; educational years: 13.3 ± 2.3). The MMSE and CAMS-R scores showed no significant differences between the two groups. A higher mean IES-R score was noted in the patient group; however, it did not reach a significant difference due to a wide standard deviation (Table 1).

**Table 1:** Demographic characteristics and summary of cognitive tests.

Characteristic	Breast Cancer Patients After Chemotherapy (n = 19)		Healthy Controls (n = 20)		p-value
	Mean or Count	SD	Mean or Count	SD	
Age (years)	43.8	6.4	50.1	2.5	0.001
Education (years)	13.9	2.2	13.3	2.3	0.435
Breast cancer stage (0, I, II, III, IV)	(0, 2, 14, 3, 0)	N/A	N/A	N/A	N/A
Chemotherapeutic drugs (docetaxel and epirubicin)	19	N/A	N/A	N/A	N/A
MMSE	28	1.283	28.316	1.453	0.508
CAMS-R	33.882	4.471	33.895	3.972	0.993
IES-R	15.941	24.055	7.079	10.498	0.187

Abbreviations: MMSE: Mini-Mental State Examination.

CAMS-R: Cognitive and Affective Mindfulness Scale-Revised.

IES-R: Impact of Event Scale-Revised.

SD: standard deviation.

N/A: not applicable.

### mfALFF Analysis

**Figure 1:**
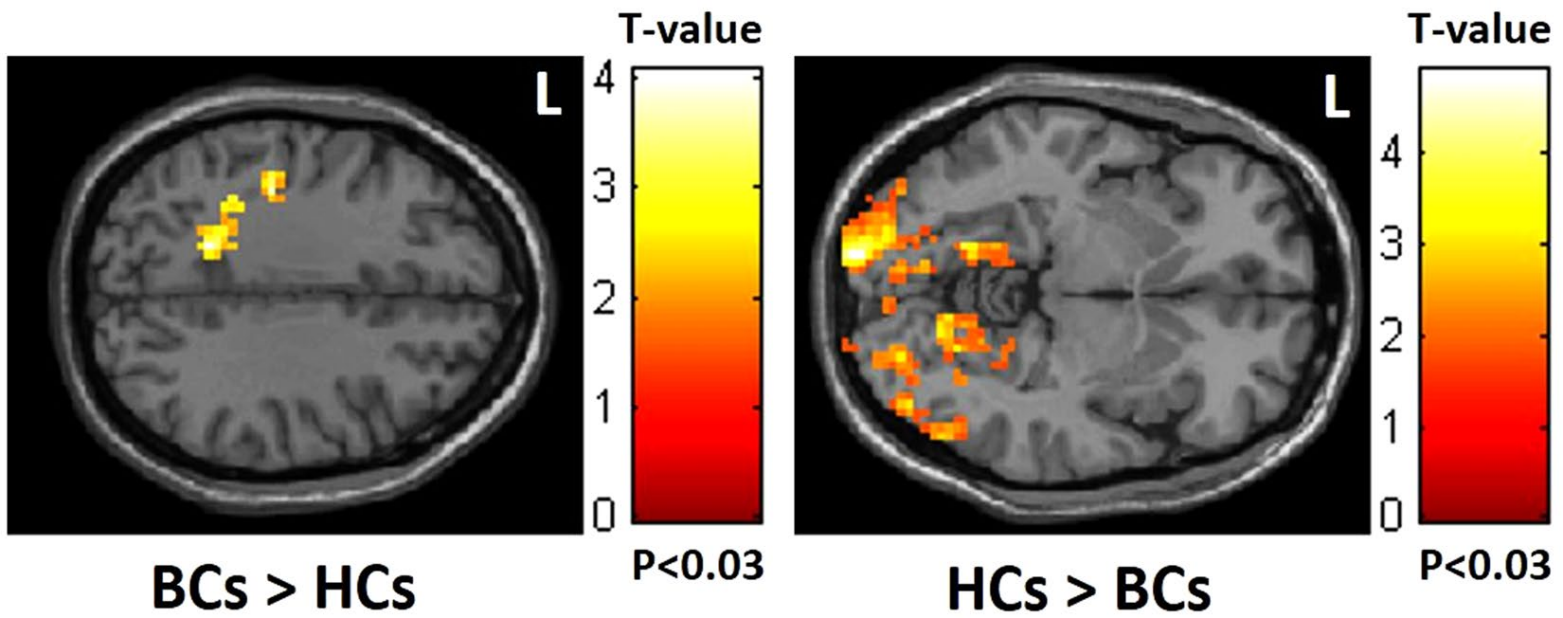
Increased mfALFF in the frontoparietal lobe but decreased mfALFF in the occipital lobe in BCs compared to HCs. (BCs: breast cancer; HCs: healthy controls).

Compared with the healthy controls, altered brain functional connectivity in the dorsal attention network (DAN) was noted in the patients with breast cancer who showed decreased mfALFF in the occipital lobe but increased mfALFF in the frontoparietal lobe (Fig. 1).

### Cognitive Tests Correlation Analysis

**Figure 2:**
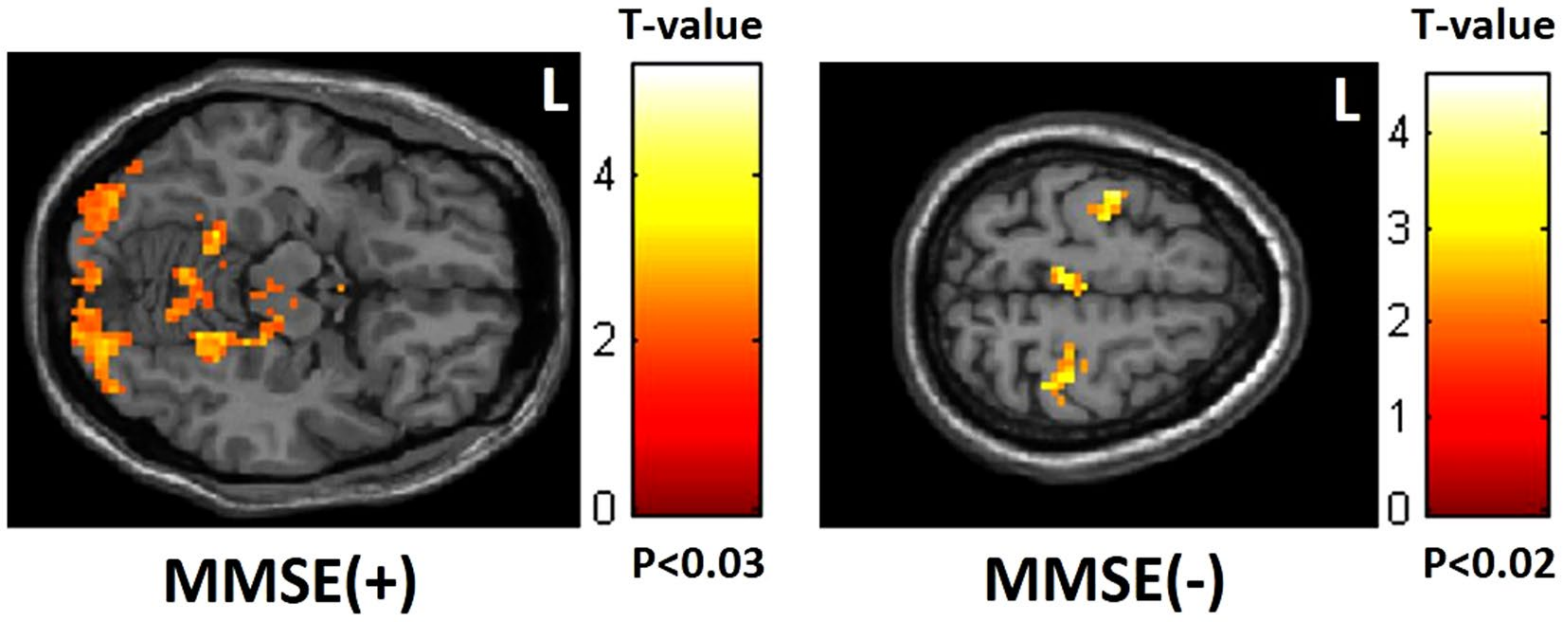
Positive correlation between MMSE scores and mfALFF in the occipital lobe but negative correlation in the frontoparietal lobe. (MMSE: Mini-Mental State Examination).

**Figure 3:**
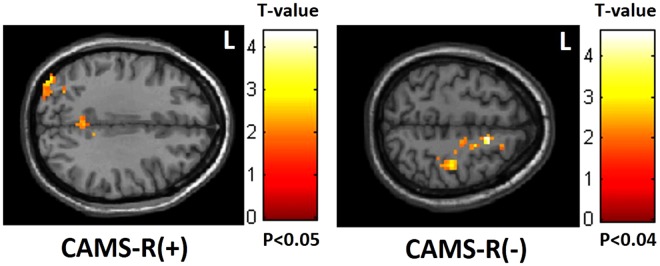
Positive correlation between CAMS-R scores and mfALFF in the occipital lobe but negative correlation in the frontoparietal lobe. (CAMS-R: Cognitive and Affective Mindfulness Scale-Revised).

**Figure 4:**
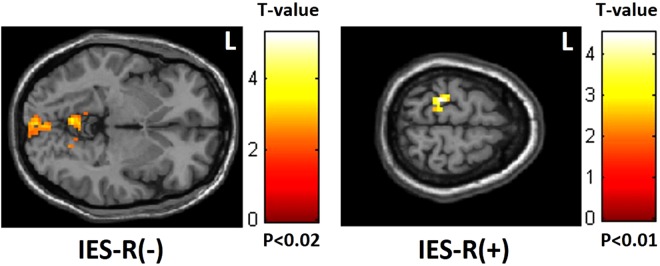
Negative correlation between IES-R scores and mfALFF in the occipital lobe but positive correlation in the frontoparietal lobe. (IES-R: Impact of Event Scale-Revised).

The MMSE and CAMS-R scores showed a positive correlation with mfALFF in the occipital lobe but a negative correlation in the frontoparietal lobe (Figs 2 and 3). By contrast, IES-R scores showed a positive correlation with mfALFF in the frontoparietal lobe but a negative correlation in the occipital lobe (Fig. 4).

## Discussion

In this cross-sectional study, we used rs-fMRI with the mfALFF approach to investigate and compare the effects of chemotherapy between female patients with breast cancer and sex-matched healthy controls. In addition, we evaluated the correlation between brain functional connectivity alterations and clinical assessments of cognitive function. First, we found alterations of the DAN among the patients with breast cancer who showed increased mfALFF in the frontoparietal lobe but decreased mfALFF in the occipital lobe compared with the healthy controls. Second, correlations were observed between MMSE and CAMS-R scores with mfALFF, which showed a positive correlation in the occipital lobe but a negative correlation in the frontoparietal lobe. By contrast, IES-R scores showed a positive correlation with mfALFF in the frontoparietal lobe but a negative correlation in the occipital lobe.

The concept of anatomical and functional attention networks in the human brain was first introduced by Corbetta and Shulman *et al*. and can be separated into dorsal and ventral systems^57^. The DAN is involved in the top-down, voluntary control of attention, driving from the frontoparietal lobe and reacting at the occipital lobe. The ventral attention network (VAN) is involved in detecting unattended or unexpected stimuli and triggering shifts of attention. Moreover, dorsal and ventral systems have flexible interactions with each other^58^. A recent study using arterial spin labeling perfusion evaluated breast cancer patients 1 month after chemotherapy and compared their findings with those of both patients with breast cancer before chemotherapy and healthy controls; the results showed significant increases in cerebral blood flow due to overcompensatory mechanisms in multiple brain areas associated with altered attention networks^59^. Another longitudinal study used task-based fMRI and found decreased functional connectivity in the DAN 1 month after chemotherapy, suggesting that changes in connectivity in the DAN have implications for understanding the effect of chemotherapy on cognitive performance^32^.

Fox *et al*. first used rs-fMRI to evaluate attention networks and found that the functional organization of attention networks could be represented in the correlation structure of spontaneous activity, and the intrinsically defined attention networks were broadly consistent with the task-based model^60^. Using rs-fMRI with SCA in breast cancer patients after chemotherapy evaluation, several recent studies showed alterations of brain functional connectivity frequently found in the frontal lobe and parietal lobe that support attention and executive function^39,40,61,62^. A recent study combined rs-fMRI and DTI to evaluate breast cancer patients after chemotherapy and found a decrease in Regional Homogeneity (ReHo), mainly in the frontal lobe, and a decrease in fractional anisotropy (FA) in the superior fronto-occipital fasciculus, a part of a widely distributed visuospatial attention network^63^. Other diseases associated with cognitive dysfunction, such as type 2 diabetes mellitus (T2DM) and Alzheimer’s disease (AD), also showed disrupted attentional networks through the use of rs-fMRI approaches^64,65^. Furthermore, several recent studies have suggested that when functional alterations occur, the brain has the capacity for compensatory activation that allows for the recruitment of alternate brain regions to maintain cognitive performance^13,66^. Compensatory activation has been observed in cancer survivors due to requiring more effort during cognitive tasks, and cancer survivors are more easily disrupted in the real world of distractions^32,67^. Thus, we hypothesized that our results showed alterations of the DAN among the patients with breast cancer who showed increased mfALFF in the frontoparietal lobe but decreased mfALFF in the occipital lobe compared with the healthy controls may be explained by compensatory mechanisms. By contrast, we did not find a significant difference in VAN between the patients with breast cancer and healthy controls. Attention network studies in T2DM and AD using rs-fMRI showed similar results with fewer significant correlations between VAN and cognitive performance^64,65^. Therefore, we suggest that VAN may be more stable and less susceptible to the effect of breast cancer after chemotherapy treatment compared with DAN.

In our cognitive test correlation analysis, we found a negative correlation of mfALFF in the frontoparietal lobe but a positive correlation of mfALFF in the occipital lobe with both MMSE and CAMS-R scores (both contented attention domains), which indicated that DAN may play a crucial role in cognitive function. Recent studies have enhanced the role of alteration of attention in cancer-associated cognitive impairments and suggested that cancer survivors’ perception of memory problems is related to deficits in earlier stages of information processing related to attention rather than memory^22,68^. Notably, we also found that IES-R scores showed a positive correlation with mfALFF in the frontoparietal lobe but a negative correlation in the occipital lobe, which indicated that the alterations of DAN may be associated with psychological distress. The findings of MRI structure analysis performed by McDonald *et al*. suggested that alterations are primarily related to the effects of chemotherapy rather than the cancer disease process or effects of other cancer treatments^23^. However, several recent clinical and MRI studies have suggested that cognitive alterations, including compromised attention function, can exist in patients with breast cancer before chemotherapy, which may contribute to PTSD^15–19^. PTSD symptoms affected more than 80% of patients with breast cancer after the disease was diagnosed and even before any treatment and could last more than 1 year^69^. Taken together, we suggest that the effects of chemotherapy treatment and psychological distress may both play important roles in DAN alterations in patients with breast cancer within 6 months after chemotherapy.

This study has several limitations. First, the sample size used was relatively small, which may have caused the differences in age between the groups. The consistent chemotherapeutic agent was used, and age was added as a covariant factor in our patients with breast cancer, which may help diminish this issue. However, the correlations should be explained with caution, and future research involving a larger sample size of patients with breast cancer is recommended^70^. Second, the cross-sectional design did not allow us to observe the effects of both chemotherapy and psychological stress on different time periods in breast cancer participants. Hence, longitudinal studies are needed to examine these effects.

## Conclusions

By using rs-fMRI correlated with cognitive tests, we revealed alterations of mfALFF in DAN in patients with breast cancer within 6 months after chemotherapy compared with healthy controls. The potential underlying mechanisms may be related to the effects of both chemotherapy and psychological distress.
